# Calculus detection technologies: where do we stand now?

**Published:** 2014

**Authors:** V Archana

**Affiliations:** * Department of Periodontics, PMS College of Dental Science, Thiruvananthapuram, Kerala, India

**Keywords:** biofilm, calculus, periodontal disease, scaling, root planing

## Abstract

Epidemiological studies have implicated dental calculus as an ideal substrate for subgingival microbial colonization. Therefore, the main objective of periodontal therapy is to eliminate the microbial biofilm along with the calculus deposits from the root surface by root surface debridement. Over the past years, a large number of clinical and laboratory studies have been conducted to evaluate the efficacy of calculus removal by various methods. None of these conventional methods or devices was effective in completely eliminating all the calculus from the diseased root surfaces. In this context, a number of newer technologies have been developed to identify and selectively remove the dental calculus. Regarding this fact, the present article highlights a critical review of these devices based on published clinical and experimental data.

## Introduction

Periodontal disease is a common, complex multifactorial disease characterized by the destruction of periodontal tissues and loss of connective tissue attachment. Bacterial plaque is the main etiological factor implicated in the etiology of periodontal disease. So, the removal of this bacterial biofilm is a decisive factor in the prevention as well as the treatment of this disease [**1**].

Dental calculus represents the mineralized bacterial plaque and is covered by an unmineralized viable bacterial plaque [**2**]. There is mounting evidence in the literature that dental calculus provides an ideal porous niche for plaque retention and subsequent mineralization. Therefore, it has been considered as a secondary etiological factor in periodontitis [**3**]. So, the main objective of the conventional cause-related therapy is the removal of these bacterial biofilms along with the calculus deposits, in order to maintain a biologically compatible root surface [**4**].

The proper evaluation of a smooth and clean root surface is essential to enable a thorough and substance-sparing debridement. Clinicians are often uncertain about the nature of the subgingival root surface while performing periodontal instrumentation. In this context, the past few years have witnessed the development of several calculus-detection technologies to support the clinician’s decision to either stop or continue the root surface debridement.

**Significance of dental calculus for the periodontal disease process**

There have been extensive cross-sectional and longitudinal studies showing the significance of calculus in the initiation and progression of periodontal disease [**5**]. However, the design of these studies failed to establish a cause and effect relationship.

It has been debated whether or not calculus may exert a detrimental effect on the periodontal tissues due to its rough surface. However, as calculus is always covered with a viable bacterial plaque, it is difficult to distinguish between the effects of calculus or plaque on the periodontal tissues [**6**]. In fact, the early studies in experimental animals reported that autoclaved calculus does not cause a pronounced inflammation or abscess formation in the connective tissues [**7**] and also provided evidence that a normal epithelial attachment can be formed on the calculus surface which has been treated with chlorhexidine [**8**]. These studies clearly exclude the possibility of dental calculus as a primary cause of periodontal diseases. The effect of calculus seems to be secondary by providing an ideal surface configuration conducive to further plaque accumulation and subsequent mineralization.

Calculus deposits may have developed in areas with difficult access for the oral hygiene or may jeopardize proper oral hygiene practices due to the size of the deposits. Calculus may also amplify the effects of bacterial plaque by keeping the bacterial deposits in close contact with the tissue surface, thereby influencing both the bacterial ecology and the tissue response [**9**].

Several animal and clinical studies have shown that the diligent and complete removal of the subgingival plaque on the top of the subgingival calculus will result in the healing of periodontal lesions and maintenance of healthy gingival and periodontal tissues. These studies have clearly elucidated the role of subgingival calculus as plaque-retentive factor [**10**,**11**].

**Conventional calculus detection technologies**

The traditional subgingival root debridement procedure consists of a systematic treatment of all the diseased root surfaces by using hand, sonic and / or ultrasonic instruments followed by tactile perception until the root surface feels smooth and clean. However, the traditional tactile perception of the subgingival root surface without the visual accessibility lacks sensitivity, specificity and reproducibility. Thus, the subgingival debridement may lead to varying degrees of residual calculus, removal of root cementum or both [**12**,**13**]. In order to overcome these shortcomings, a number of different technologies have been incorporated into dental devices for the purpose of identifying and selectively removing the dental calculus.

**Recent advances in calculus detection technology**

Current technologies for calculus detection comprise detection-only systems as well as combined calculus-detection and calculus-removal systems (**Table 1**).

**Table 1:** Calculus detection technology

Treatment goal	Technology	Device name
Calculus detection only	Fiberoptic endoscopy	Perioscopy
	Spectro-optical technology	Detectar
	Autofluorescence	Diagnodent
Combined calculus detection	Ultrasound	Perioscan
& removal	Laser & autofluorescence	Keylaser3

**Detection-only systems**

**a. Fiberoptic endoscopy- based system**

The fiberoptic endoscopy based technology for calculus detection is currently being used in only one device, Perioscopy (Perioscopy Inc., Oakland, CA, USA). Perioscopy is a minimally invasive miniature periodontal endoscope and is inserted into the periodontal pocket for subgingival visualization of the root surface at magnifications of 24–48x. This system consists of a 10,000-pixel fiberoptic bundle with 1mm diameter surrounded by multiple illumination fibers, a light source, an irrigation system and a display monitor with liquid crystal.

This automated system aids in the visualization of the subgingival root surface, tooth structure and residual calculus in real time. Also, the magnified images can be viewed on the monitor in real time and images as well as videos can be saved in computer files. In addition, this endoscope-based system may help in identifying and locating the residual calculus spots during instrumentation. One common problem reported in studies comparing the endoscopic technique with the conventional explorer is the additional training period of at least 8hr to learn the procedure and subsequent practical experience up to 4 weeks [**14**,**15**].

Avradopoulos et al. conducted the first clinical study by treating non-responding periodontal sites (probing depth 5–8 mm) by subgingival root debridement with or without the use of the dental endoscope. No significant reductions in the pocket depth were observed in either group after treatment. Moreover, a rather long treatment time of 45 min per experimental site was noted for the endoscopy procedure [**16**].

Subsequently, a randomized clinical study was conducted by Geisinger et al. to evaluate the percentage of residual calculus in single-rooted teeth after the extraction. The teeth were treated by hand and ultrasonic instruments until the root surface was found to be clean, as assessed by either an explorer or the periodontal endoscope. After the extraction, a higher percentage of the residual calculus covering the root surface was detected microscopically in the explorer group than in the endoscope group. The difference was statistically significant only in deeper pockets and in interproximal sites (pocket depth > 6 mm) compared with the buccal sites (pocket depth > 4 mm) [**17**].

On the contrary, Michaud et al. conducted a study in molars and reported a less residual calculus covering the root surface in the endoscopy group compared with the scaling and root planing group. No differences in the residual calculus were found in deep pockets, furcation areas or on buccal/lingual surfaces. Only interproximal pockets with a depth of < 6 mm had a significantly less residual calculus in the endoscope group compared with the scaling and root planing group [**18**].

In a recent study, Wilson et al. evaluated the histologic response to the removal of calculus and biofilm with the aid of the dental endoscope. Histological evidence reported the formation of a long junctional epithelium, bone repair and no signs of chronic inflammation. However, a control group that received scaling and root planing alone was not compared with the test group [**19**].

In summary, only one clinical study to date has evaluated the clinical effects after the application of dental endoscopy. No differences were observed in pocket depth reduction between scaling and root planing alone and endoscope-aided scaling and root planing. The histological healing observed after the endoscope-aided scaling and root planing was not compared with scaling and root planing alone in a randomized clinical study. The microscopic analysis of the root surfaces after endoscopy-aided scaling and root planing reported a small benefit only in interproximal sites, in particular in single-rooted teeth with deep pockets and in multi-rooted teeth with relatively shallow pockets. Moreover, studies have shown that the fiberoptic device needs additional treatment time and operator skill compared to scaling and root planing.

**b. Spectro-optical technology based system**

The spectro-optical technology to detect calculus is currently being used in one device, DetecTar (Dentsply Professional, York, PA, USA). This automated system consists of a light-emitting diode, an optical fiber, computer and this device available as a portable cordless handpiece with a curved periodontal probe that has millimeter markings to measure pocket depths. When the subgingival calculus is irradiated by red light, it results in the production of a characteristic spectral signature caused by absorption, reflection and diffraction. These spectral signals are sensed by an optical fiber and converted into an electrical signal that is analyzed by a computer device. This device aids in the scanning of the subgingival root surface without any tactile pressure. As soon as calculus is detected, the operator receives the information by audible and luminous signals [**20**].

Krause et al. conducted an *in vitro* study to evaluate the subgingival calculus detection potential of DetecTar in 20 freshly extracted teeth affected by periodontitis. Teeth were scanned with different working-tip angulations of the fiberoptic (0, 10, 45 or 90 degrees) in the presence of different ambient fluids (blood and saline solution) and the results were compared with clinical and histological findings. The results showed that the specificity is 100% in blood and 95-100% in saline solution for all angulations. The sensitivity in saline solution was nearly 100% for all angulations and in blood, the sensitivity decreased with smaller tip angulations (100% sensitivity for 90°, 89% for 45° and 70% for 10° to 0° angulations). The combination of saline solution and a working-tip angulation of 90° resulted in the most accurate measurements [**21**].

Recently, Kasaj et al. conducted the first clinical study to assess the utility of the spectro-optical technology for subgingival calculus removal by treating a total of 44 teeth (176 surfaces). The untreated control group were scanned *in vivo* by using the DetecTar. In the treatment group, scaling and root planing was continued until no positive signal was elicited. Clinical findings were recorded by visual and microscopic examination after tooth extraction. The control group showed a sensitivity of 79.4% and a specificity of 95.1%. In the test group, out of 58 tooth surfaces that initially showed calculus and which were consequently treated until they tested negative for calculus, 10 (17%) remained partly covered with calculus, whereas 48 (83%) were completely calculus-free. In fact, nine (41%) of the 22 surfaces that were initially identified as calculus-free, harbor calculus when left untreated [**22**].

Taken together, the ability of the spectro-optical technology for calculus detection has not yet been thoroughly investigated. Therefore, the effectiveness of spectro-optical device needs to be evaluated in clinical settings.

**c. Autofluorescence based system**

Several *in vitro* studies have shown that oral microorganisms and their metabolites like porphyrins, metalloporphyrins and other chromatophores contain the fluorophores that are emitted from the dental calculus and carious lesions [**23**-**25**]. This ability of calculus to emit fluorescent light following irradiation with light of a certain wavelength enables the detection of calculus. Based on this autoflurescent property of calculus, a newer diagnostic instrument has been developed, the Diagnodent (KaVo, Biberach, Germany). The device was initially launched for caries diagnosis. Later, the device was modified to enable calculus detection.

Diagnodent is able to measure wide range of fluorescence intensities, transformed and shown on a digital display as relative calculus-detection values from 0-99 (**Table 2**) [**20**]. Readings corresponding to the calculus are indicated by a beep with an increasing sound frequency as the display value increases.

**Table 2:** Translation of Diagnodent readings to clinical conditions

Diagnodent readings	Clinical inference
≥40	Mineralized deposits
5-40	Very small calcified plaque sites or residual calculus following partial cleaning
≤5	Clean root surface

Krause et al. conducted the first *in vitro* study in surfaces of periodontally involved extracted teeth by using the Diagnodent. The teeth surfaces, partly covered with calculus and moistened with saline solution or blood, were scanned by using the automated device. The fluorescence was measured at all teeth and reproducibility was tested. The findings reported that the presence of calculus was significantly correlated with a higher intensity of fluorescence, which was not influenced by the presence of fluid. Additionally, high reproducibility for measurements after 6 and 24 h could be observed [**26**]. 

Subsequently, Folwaczny et al. conducted a study on a mannequin model to compare the effectiveness of hand instrumentation when supported by the autofluorescence-based system and conventional explorer. Forty periodontally involved extracted teeth were treated with conventional Gracey curettes until a clean root surface was obtained. In multirooted teeth, autofluorescence-based system detected a significantly smaller total area covered with residual calculus than the conventional explorer. However, both study groups reported a comparable amount of residual calculus in single-rooted teeth [**27**].

Altogether, autofluorescence-based system for calculus detection has been evaluated only *in vitro* studies so far. When used in vitro, this automated system could differentiate between calculus and cementum with high reproducibility. In a preclinical situation, the superior effect of this system could be observed only on molars. So, the diagnostic potential of the autofluorescence-based system needs to be evaluated in the clinical setting.

**Combined calculus-detection and calculus-removal systems**

Although detection-only systems demonstrated high sensitivities and specificities for the detection of calculus, the superior clinical outcomes following periodontal therapy has not been thoroughly investigated. A major disadvantage of the stand-alone diagnostic devices lies in the need to alternate between detection and debridement. In transition from a diagnosis device to a debridement instrument, information regarding the area the residual calculus is located may be lost and subsequently lead to over or under-instrumentation. The combined detection and treatment device aims to overcome this problem.

**a.Ultrasonic technology based system**

The ultrasonic calculus-detection device is based on a conventional piezo-driven ultrasonic scaler [**28**]. The ultrasonic based technology is currently available in Perioscan (Sirona, Bensheim, Germany), which provides a diagnosis mode to detect calculus deposits and a treatment mode for the conventional ultrasonic debridement at different power levels. When the ultrasonic tip touches the tooth surface, it produces different light signals both in the handpiece and in a display of the table unit. The presence of green light indicates cementum and blue light indicates calculus. An additional acoustic signal sounds, when the calculus is detected.

The advantage of the ultrasonic calculus-detection technology is that the diagnosis and treatment modes can be used successively on the surface of the same tooth. Additionally, the calculus deposits and biofilm can be removed with high and low power setting respectively.

Meissner et al. evaluated the calculus-detection capacity of a prototype of the ultrasonic device under different laboratory conditions. The calculus detection results were compared with visual findings on calculus and cementum surfaces both in a static state as well as during the movement of the probing tips. The static test reported a sensitivity of 75% and specificity of 82% and during movements of the probing tip obtained a sensitivity of 88% and a specificity of 76% [**29**,**30**].

Subsequently, Meissner et al. conducted an in vitro study to evaluate the calculus detection limit of ultrasonic device by gradually removing calculus from 50 extracted teeth until the system stopped discriminating the calculus deposits. It could be shown that the ultrasonic device is able to detect calculus deposits with a diameter of 0.2 mm with a sensitivity of 73% and a specificity of 80% [**31**].

Recently, Meissner et al. conducted the in vivo randomized clinical study to evaluate the accuracy of Perioscan to detect calculus. Teeth were scanned in situ and the detection results were compared with the visual findings after extraction. A prevalence of calculus of 22.3% was found on the scanned surfaces, and calculus and cementum were distinguishable with a sensitivity of 91% and a specificity of 82% [**32**].

In summary, various *in vivo* and *ex vivo* studies have reported the combined calculus detection and removal ability of the ultrasound technology. However, the long-term clinical outcome remains to be investigated.

**b.Laser technology based system**

Studies have shown that among the various types of lasers, Er:YAG laser has been considered the most promising for periodontal therapy [**33**,**34**], mainly due to its property to ablate soft and hard tissue without major thermal side effects. There is extensive evidence in literature to prove the comparable calculus removal effect of Er:YAG lasers (Keylaser 1 or 2; Kavo, Biberach, Germany) to conventional root debridement [**33**,**35**,**36**].

Keylaser 3 (Kavo, Biberach, Germany) is the only commercially available device, which combines calculus detection and removal in a feedback-controlled manner. The automated device contains a 655-nm InGaAs diode laser for calculus detection and a 2940-nm Er:YAG laser for calculus removal. The Er:YAG laser is activated only when the threshold value for the diode laser exceeds 0-99. As soon as the reading falls below the threshold value, the treatment laser turns off. This mechanism of Keylaser 3 helps to optimize the calculus removal and reduces the side effects associated with Er:YAG laser.

One of the earliest *in vitro* studies by Krause et al. reported that the amount of residual calculus depended on the laser fluorescence threshold levels and the laser-treated residual cementum was significantly thinner than the untreated residual cementum [**37**]. Schwarz et al. conducted the initial clinical study to compare the effect of Er:YAG laser to hand instruments and reported that the treatments with the Er:YAG laser were comparable to those obtained by hand instruments [**38**]. Subsequently, Schwarz et al. demonstrated that the treatment with the feedback controlled Er:YAG laser resulted in significantly less residual calculus and less root-surface alterations than hand instrumentation [**39**].

Derdilopoulou et al. conducted a study to evaluate the microbiological findings after the periodontal therapy by using curettes, Er: YAG laser, sonic and ultrasonic scalers. The results showed that all four treatment modalities resulted in a significant reduction of Porphyromonas gingivalis, Prevotella intermedia, Tannerella forsythia and Treponema denticola after 3 months. Laser and sonic instrumentation failed to reduce Aggregatibacter actinomycetemcomitans significantly [**40**]. Sculean et al. and Tomasi et al. reported no statistically significant differences between the feedback controlled Er.YAG laser debridement and the ultrasonic treatment [**41**,**42**].

Altogether, there is evidence in literature that laser-based detection and treatment of calculus are able to effectively remove subgingival calculus and prevent the unwanted removal of root substance. However, the results were comparable with hand and ultrasonic instrumentation and need long-term clinical trials to prove the efficiency.

## Conclusion

Taken together, the published data on new technology-assisted treatments are only available from laboratory research results and have yet to show clinical superiority in comparison with conventional systems. Therefore, more randomized clinical trials should be conducted to further explore the impact of these newer devices to improve long-term treatment outcome.

**Source of Funding:** None

**Disclosures:** None to declare
